# Cryptic behavior and activity cycles of a small mammal keystone species revealed through accelerometry: a case study of Merriam’s kangaroo rats (*Dipodomys merriami*)

**DOI:** 10.1186/s40462-023-00433-x

**Published:** 2023-11-02

**Authors:** Ryan J. Hanscom, Jessica L. Hill, Charlotte Patterson, Tyler Marbach, Jeet Sukumaran, Timothy E. Higham, Rulon W. Clark

**Affiliations:** 1 Department of Biology, San Diego State University, San Diego, CA USA; 2 Department of Evolution, Ecology, and Organismal Biology, University of California, Riverside, CA USA; 3 Department of Forestry and Wildlife Management, Inland Norway University of Applied Sciences, Koppang, Norway

**Keywords:** Biologging, Ecosystem engineer, Foraging, Moonlight, Heteromyid

## Abstract

**Background:**

Kangaroo rats are small mammals that are among the most abundant vertebrates in many terrestrial ecosystems in Western North America and are considered both keystone species and ecosystem engineers, providing numerous linkages between other species as both consumers and resources. However, there are challenges to studying the behavior and activity of these species due to the difficulty of observing large numbers of individuals that are small, secretive, and nocturnal. Our goal was to develop an integrated approach of miniaturized animal-borne accelerometry and radiotelemetry to classify the cryptic behavior and activity cycles of kangaroo rats and test hypotheses of how their behavior is influenced by light cycles, moonlight, and weather.

**Methods:**

We provide a proof-of-concept approach to effectively quantify behavioral patterns of small bodied (< 50 g), nocturnal, and terrestrial free-ranging mammals using large acceleration datasets by combining low-mass, miniaturized animal-borne accelerometers with radiotelemetry and advanced machine learning techniques. We developed a method of attachment and retrieval for deploying accelerometers, a non-disruptive method of gathering observational validation datasets for acceleration data on free-ranging nocturnal small mammals, and used these techniques on Merriam’s kangaroo rats to analyze how behavioral patterns relate to abiotic factors.

**Results:**

We found that Merriam’s kangaroo rats are only active during the nighttime phases of the diel cycle and are particularly active during later light phases of the night (i.e., late night, morning twilight, and dawn). We found no reduction in activity or foraging associated with moonlight, indicating that kangaroo rats are actually more lunarphilic than lunarphobic. We also found that kangaroo rats increased foraging effort on more humid nights, most likely as a mechanism to avoid cutaneous water loss.

**Conclusions:**

Small mammals are often integral to ecosystem functionality, as many of these species are highly abundant ecosystem engineers driving linkages in energy flow and nutrient transfer across trophic levels. Our work represents the first continuous detailed quantitative description of fine-scale behavioral activity budgets in kangaroo rats, and lays out a general framework for how to use miniaturized biologging devices on small and nocturnal mammals to examine behavioral responses to environmental factors.

**Supplementary Information:**

The online version contains supplementary material available at 10.1186/s40462-023-00433-x.

## Background

Small mammals are among the most abundant vertebrates in many terrestrial ecosystems, providing numerous linkages between other species as both consumers and resources [1–7]. Many small mammals act as ecosystem engineers, excavating extensive burrow systems that impact vegetation and provide habitat for many other species [8–11]. Despite their importance in the broader community, we still lack detailed information on key aspects of the behavior and activity of many small mammals, as numerous species are nocturnal, secretive, semi-fossorial, and thus difficult to study using traditional observation techniques.

Detailed fine-scale data on the activity and behavior of keystone species represents an important component of community ecology, as this information provides invaluable insight into interactions with other species and broader ecological niche parameters [12–14]. However, many methods used to quantify behavior, such as direct observation, are limited by expense, time, animal crypsis, physical or temporal barriers, or the impact of a human observer on the natural expression of behavior [15, 16]. In recent years, some of these shortcomings have been addressed by advancements in biologging technology that use miniaturized animal-borne tags to collect data about movement, behavior, physiology, and/or abiotic conditions [17–19]. These novel *next generation natural history* biologging techniques provide a much deeper understanding of the behavior and ecology of species that are both easy to observe in nature and ones that are not amenable to direct observation [18, 20].

The use of accelerometers to quantify behavior and activity has its own set of limitations, including the need to create detailed validation datasets, relatively crude classifications of behavioral states, the lack of broader ecological context for behavioral expression, and the inability of smaller species to carry large enough devices for long enough periods of time [17]. These limitations have led to taxonomic bias in the use of accelerometry, with most studies being restricted to larger-bodied mammals, birds, and charismatic megafauna that are typically diurnal or crepuscular (and thus easier to observe for validation [17]). However, statistical approaches for analyzing animal movements continue to be refined using both supervised and unsupervised machine learning (reviewed in Hoffman et al. [21]), and the continued miniaturization of biologgers is creating opportunities for studying smaller-bodied species.

To date, the smallest mammals that have been studied using advanced biologging techniques to quantify activity and behavioral patterns are Western chipmunks (*Tamias alpinus* and *T. speciosus*), diurnal sciurid rodents weighing ~ 50 g. Hammond et al. [22] attached miniaturized accelerometers (1.5–2.5 g) for approximately 2.5 d per individual to measure activity patterns. This study established a general framework for using animal-borne accelerometry on small-bodied animals, but there have not yet been any studies on smaller bodied species that quantify more fine-scaled behavioral states (other than just active versus inactive) or deploy logging devices over longer periods of time. In contrast, the use of accelerometers to study the behavior of large mammals has provided insight on not only activity patterns, but also details of movement type, hunting behaviors, and energy expenditure (e.g., [23–25]).

Here, we use animal-borne accelerometry to classify the behavior of free-ranging kangaroo rats (*Dipodomys merriami*), a nocturnal heteromyid rodent (< 50 g) that is the most abundant terrestrial mammal in many arid regions of North America [26]. Kangaroo rats are semi-fossorial and granivorous, storing and dispersing plant seeds while digging extensive burrow systems. Numerous studies have shown that kangaroo rats can have profound effects on their broader community, acting as both keystone species and ecosystem engineers [27–30]. Additionally, they are a major prey item for several predators at higher trophic levels such as coyotes and foxes [31, 32], rattlesnakes [3, 6], and birds of prey [33–35]. Although there has been significant research on kangaroo rat activity and foraging patterns, it has been limited by the inherent shortcomings associated with direct observation, and we still lack important information on many aspects of the activity and behavior of these critical species (e.g., relative levels of activity during different phases of the diel cycle and how that activity is affected by various environmental parameters).

Our study uses animal-borne accelerometry to classify the behavior of free-ranging individuals, highlighting the utility of this method for collecting comprehensive datasets on the behavior and activity of secretive, nocturnal, and small-bodied terrestrial mammals that play critical roles in ecosystems across the globe. We developed an integrative approach using miniaturized accelerometry and radiotelemetry to identify ecologically relevant behavioral categories such as traveling and foraging. Additionally, we pioneered techniques to validate these behaviors in the field through focal observation and recording of free-ranging individuals, and then explored how different daily and nightly light phases and environmental variables influenced the behavior and activity cycles of Merriam’s kangaroo rats. In particular, our study allowed us to address the question of how variation in moonlight impacts kangaroo rat activity, an important question steeped in uncertainty, with a number of conflicting studies supporting either lunarphilia or lunarphobia [36–41].

## Methods

### Study site, capture procedures, and species

The study was carried out May-August 2021 at Marathon Grassland Preserve (MGP), a 1093-ha Chihuahuan desert grassland habitat located in West Texas, USA. The site was dominated by Trans-Pecos loamy plains grassland and sparse creosote scrub. We captured Merriam’s kangaroo rats (*D. merriami)* using Sherman live traps and marked captured individuals with 10 mm Biomark pit-tag injected subcutaneously on the dorsum. Kangaroo rats were weighed to the nearest gram, and individuals that were > 40 g in mass (*n* = 14; 9 females and 5 males; Additional File 1; Table S1) were fitted with a ~ 2.5 g accelerometer/Very High Frequency (VHF) package to the dorsum following a similar harnessing technique to that of Shier & Swaisgood [42]. The package included an accelerometer (model AXY 5, Technosmart Europe Srl., Rome, Italy; dimensions = 20 L x 15 W x 9 H mm; mass = ~ 1.8 g), a VHF microtransmitter (Wildlife Materials model SOM-2011; mass = 0.8 g) allowing for the relocation of individuals and removal of the accelerometer after the ~ 5-day monitoring period (range = 1–6.5 days; some deployments ended early due to early battery discharge or device attachment failure). Accelerometers were triaxial, placed in the same orientation across all individuals, and set to record forces between − 10 and 10 g_force_ at 25 hz. After attachment individuals were held for ~ 20 min to ensure good fit of bio-loggers (i.e., they were unable to roll over and remove the backpack) and then released at their point of capture. Individuals were then periodically relocated via radiotelemetry (typically twice a day) for validation recording and to verify retention of bio-loggers. If bio-loggers were still retained after 6 days, individuals were re-trapped for removal of the device and then immediately released at point of capture.

### Free-ranging behavioral observations and scoring

We used VHF radio tracking (Advanced Telemetry Systems R410 Receiver) and a handheld flexible 3-element Yagi antenna to locate tagged kangaroo rats. Once individuals were located, two observers would work together with a telephoto video camera (10X optical zoom) with infrared (IR) recording capability (Sony Handycam Model DCR-SR80) and an IR flashlight (UniqueFire, 1605 T38 850 nm; invisible to kangaroo rats) to video record behaviors of kangaroo rats at a distance over 10 m, moving slowly and carefully to avoid unnecessary disturbance. Individuals were filmed for as long as they were visible. We spent approximately 3 h per night throughout the sampling period tracking kangaroo rats and recording videos for behavioral validation when they were active on the surface. From these efforts, we scored all videos that showed clear and unambiguous examples of kangaroo rats exhibiting the behaviors described in our ethogram, resulting in 122 m of validated video observations (Additional File 1; Table S2).

Behavioral scores were time matched to accelerometer readings (to within 1 s) to generate annotated acceleration datasets by recording a video of the exact time (using the ExactTime™ software application by ©Neurovat, 2023) as the accelerometry device began logging data (Tecnosmart devices emit a visible signal when powering on). We then also recorded the ExactTime™ (©Neurovat, 2023) to the nearest second at the outset of all validation video recordings of kangaroo rats wearing accelerometry devices [43], and (as a further redundancy) whenever accelerometry devices were turned off after a deployment. All units were calibrated by Technosmart before being used in the field. Additionally, we manually examined accelerometry signatures for any evidence of clock drift when scoring validation videos of individuals many days after the onset of datalogging. We found no evidence of clock drift over the ~ 6.5 d that loggers were deployed. We initially viewed exploratory videos in order to construct a detailed ethogram and determine what potential behavioral classes could be feasibly incorporated into our modeling framework (Figure S1). Additional review determined that several behaviors (e.g., digging, kicking sand, rolling) were too rare for incorporation into the modeling framework, which led us to group behaviors into four broad classes that would be frequent enough to train a model and inclusive of key activities: motionless, traveling, foraging, and grooming (Table 1).

**Table 1:** Description and function of four kangaroo rat behaviors used for accelerometry classification

Behavior	Description	Function
Motionless	Standing (most common) or lying down while remaining still	Resting
Travel	Hopping slowly or quickly	Movement
Foraging	Head down while using forelimbs to collect, consume, or cache seeds	Caching or consumption
Grooming	Scratching or licking legs, face, or tail while standing in place	Sanitation

### Behavioral classification algorithms

We used the open-access web application AcceleRater to train classification algorithms based on our free-ranging kangaroo rat acceleration datasets [44]. First, because class imbalance can lead to inference issues when using machine-learning [45], all datasets were manually balanced so that each behavioral class was subsampled to the behavior that had the fewest samples from our free-ranging kangaroo rat acceleration datasets (in all cases, this was either the behavior class foraging or grooming). We then ran a linear support vector machine, a decision tree, and a random forest algorithm (as these models are typically used when using acceleration data to predict animal behavior) that either included all summary statistics available in AcceleRater (mean acceleration, standard deviation, maximum, minimum, vector norm, covariance, Pearson correlation, dynamic body acceleration, overall dynamic body acceleration, mean-diff, std-diff, wave amplitude, line crossings, 25 percentile, 50 percentile, and 75 percentile) or just the summary statistics of mean acceleration, standard deviation of acceleration, and overall dynamic body acceleration at what we deemed biologically relevant and distinct window sizes of 1, 2, 3, 6, and 9 s. For each unique model and window size, we trained the model via a (50/50) train-test split, where a random 50% of the data was used to train each model and the remaining 50% of the data set was used to test the model. We implemented the same methods as Clermont et al. [23] and Hanscom et al. [43] to identify which algorithm optimally classified behaviors and we calculated accuracy, precision, and recall from rates of true positives, false positives, and false negatives.

### Kangaroo rat diel activity budgets and response to moonlight and weather

**Fig. 1:**
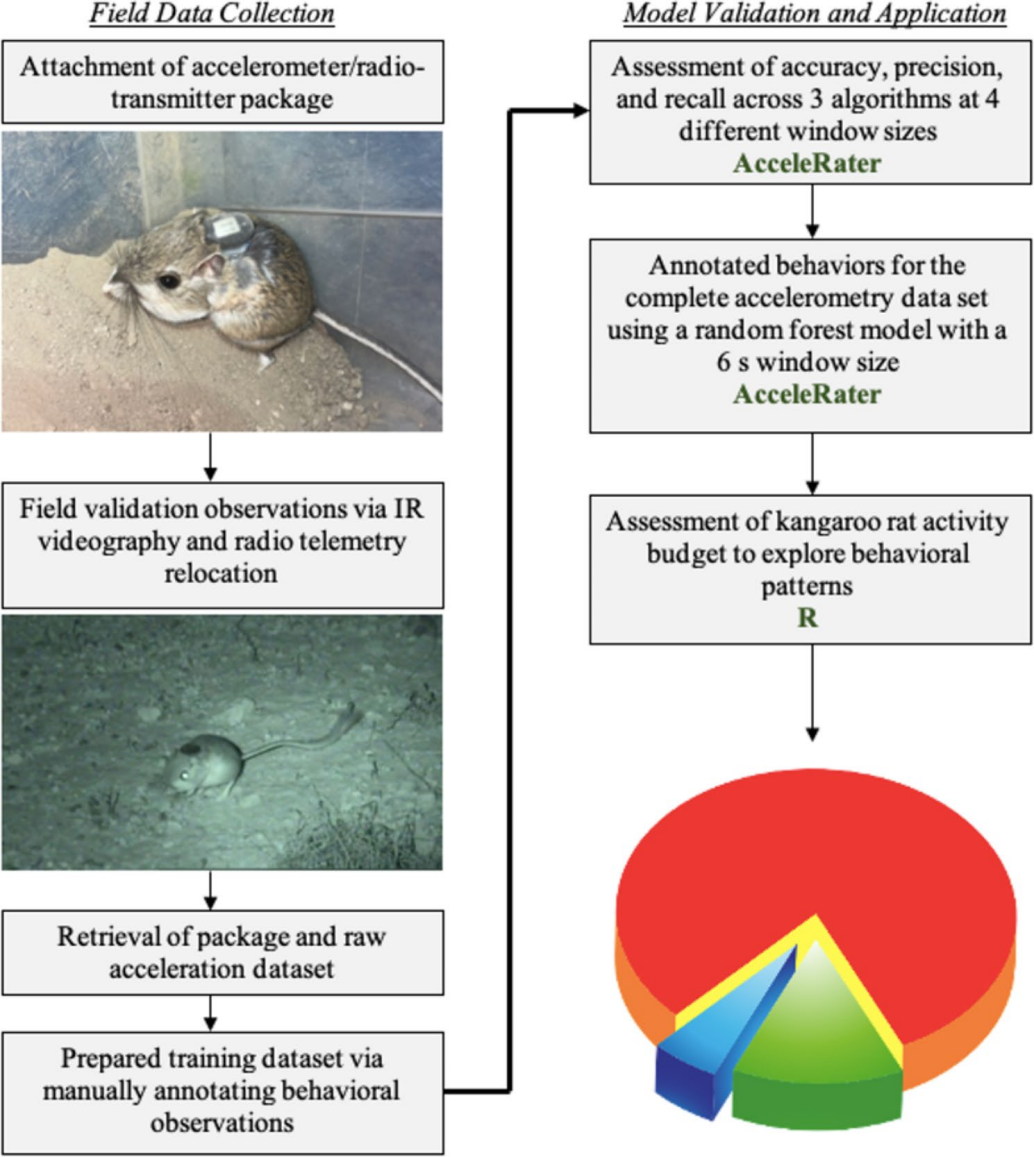
Stepwise methodological workflow for the behavioural classification of accelerometry data and the development of an activity budget using short duration, high frequency accelerometry on a small mammal < 50 g. More details of each step presented in this workflow can be found in the methods section

The stepwise methodological workflow for behavioral classification of accelerometry data and assessment of activity budgets is depicted in Fig. 1.We used the top performing algorithm from our model selection procedure to annotate our complete accelerometry dataset. We then determined kangaroo rat activity budgets by calculating proportions of time and expression for each behavior across all individuals.

**Fig. 2:**
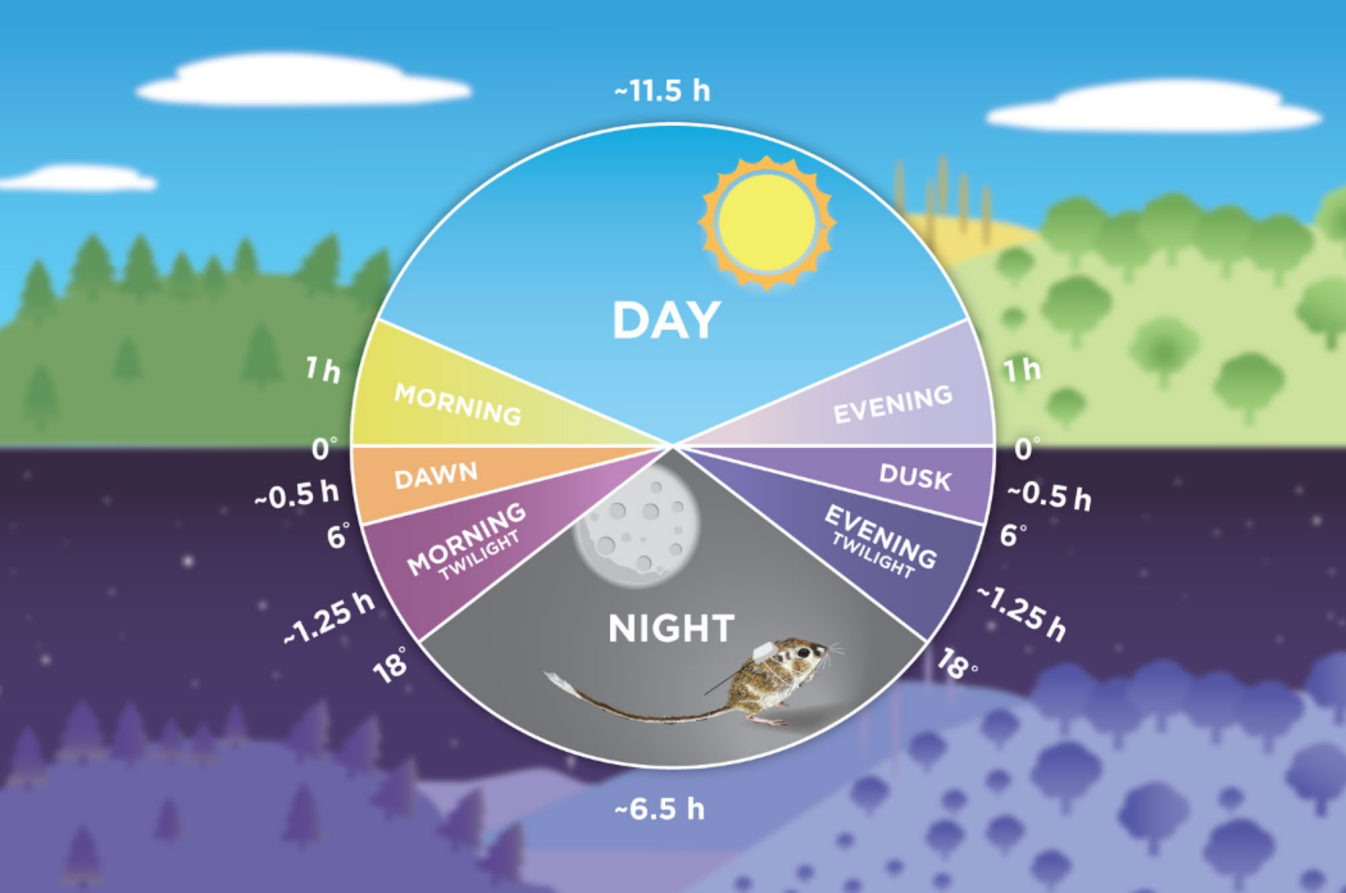
An illustration of light phases considered during the study period. Artwork for this figure was designed and produced by Alexandra Coots

Specifically, we determined behavioral activity budgets at different light phases, moon intensity and illumination, and moon position (following several previous ecological studies [46–48]). We used the *suncalc* package in R to retrieve daily light phase times, moonrise and moonset times, and sunrise and sunset times for our study site [49]. Moon illumination levels were calculated on a continuous scale ranging from 0.00 (new moon) to 1.00 (full moon). To further understand how moon illumination impacted activities, we converted moon illumination levels into a three-level categorical variable called moon intensity, where < 0.33 = low, 0.33–0.66 = medium, and > 0.66 = high [48]. The eight light phases we used are defined by the position of the sun relative to the horizon and are the following: evening (last hour above horizon; 1 h), dusk (0–6° below horizon; ~0.5 h), evening twilight (6–18° below horizon; ~1.25 h), night (> 18° below horizon; ~6.5 h), morning twilight (18–6° below horizon; ~1.25 h), dawn (6–0° below horizon; ~0.5 h), morning (first hour above horizon; 1 h), and day (above horizon minus the first and last hour sunlight; ~12 h; Fig. 2; [48]). Our study site is located in an arid and dry environment, and thus almost all nights were clear with no cloud cover. However, to be conservative, we also reran analyses after removing all nights with a precipitation rate > 0, since cloud cover is correlated with precipitation rate. Lastly, because environmental variables such as temperature, wind, and relative humidity have been shown to impact small mammal activity as estimated from trapping data [50–52], we investigated how they impacted accelerometry-based estimates of kangaroo rat behavior. We averaged weather data (average nightly air temperature, wind speed, and relative humidity) from two nearby weather stations (KTXMAR8 and KTXALPIN66; both located in similar habitat to our site and ca. 20 km straight-line distance from the field site) included in the Weather Underground network [53, 54].

We examined the effect of moonlight and environmental variables on behaviors in a generalized linear mixed effects modeling (GLMM) framework. First, we investigated the diel activity patterns of kangaroo rats by examining whether the proportion of time spent in each behavioral state (motionless, traveling, grooming, and foraging) was influenced by light phase (all eight light phases as defined above) and sex as fixed effects. Next, we examined whether the proportion of time spent in each behavioral state was influenced by moon intensity (low, medium, or high), moon position (up or down), and their interaction, as well as sex, as fixed effects. This analysis only included light phases when the sun was > 0° below the horizon (i.e., dusk, evening twilight, night, morning twilight, and dawn). Lastly, we used a GLMM to investigate how the abiotic factors of temperature, wind speed, and relative humidity affected kangaroo rat behavioral state at a nightly scale. Additionally, we included moon illumination as a nightly continuous variable in this model to corroborate any patterns found prior regarding the influence of moonlight on kangaroo rat behavior. For each behavior, we explored multiple distribution families, aiming to identify the most suitable family for modeling the data. The candidate distribution families considered were Gaussian, Poisson, Binomial, and Gamma. We employed the Akaike Information Criterion (AIC) as the primary model selection criterion, and then used the top model with the associated best family as the GLMM for each behavior (Additional File 1; Table S3). All environmental variables, as well as sex, were included as fixed effects. To avoid inferential errors associated with collinearity, we calculated the variable inflation factors (VIF) for each predictor variable. All variables had a VIF < 3; thus we retained all variables in our models [55, 56]. All GLMMs included kangaroo rat ID and date as random factors. We calculated model fit using conditional R-squared values, and significance of fixed effects was determined using Wald chi-square (*χ*^*2*^) tests [57].

## Results

### Behavioral classification using accelerometry data

The random forest model at a window size of 6 s that used all summary statistics available in AcceleRater (see methods above) produced the greatest average accuracy, precision, and recall values compared to all other algorithms across all other window sizes (Overall model accuracy = 85.3%; Additional File 1 (Table S4)). The retained model had high accuracy (> 92.5%), precision (> 85%), and recall (> 87.5%) for both behavioral classes motionless and travel. Accuracy (> 87.5%), precision (> 67.5%), and recall (> 73.0%) were somewhat lower for the behavioral classes foraging and grooming. However, when the model incorrectly classified either foraging or grooming, it typically conflated these two behaviors. Thus, when the model incorrectly classified foraging, it classified foraging as grooming 12% of the time and traveling and motionless at 0%; when the model incorrectly classified grooming as foraging at 22%, traveling at 5%, and motionless at 0%. This is likely because grooming and foraging are similar movement patterns. Consequently, our estimation of activity patterns was highly accurate when kangaroo rats were motionless versus traveling as well as when they were engaged in non-travel activity, and moderately accurate at estimating when that non-travel activity was either foraging or grooming.

### Kangaroo rat diel activity patterns

**Fig. 3:**
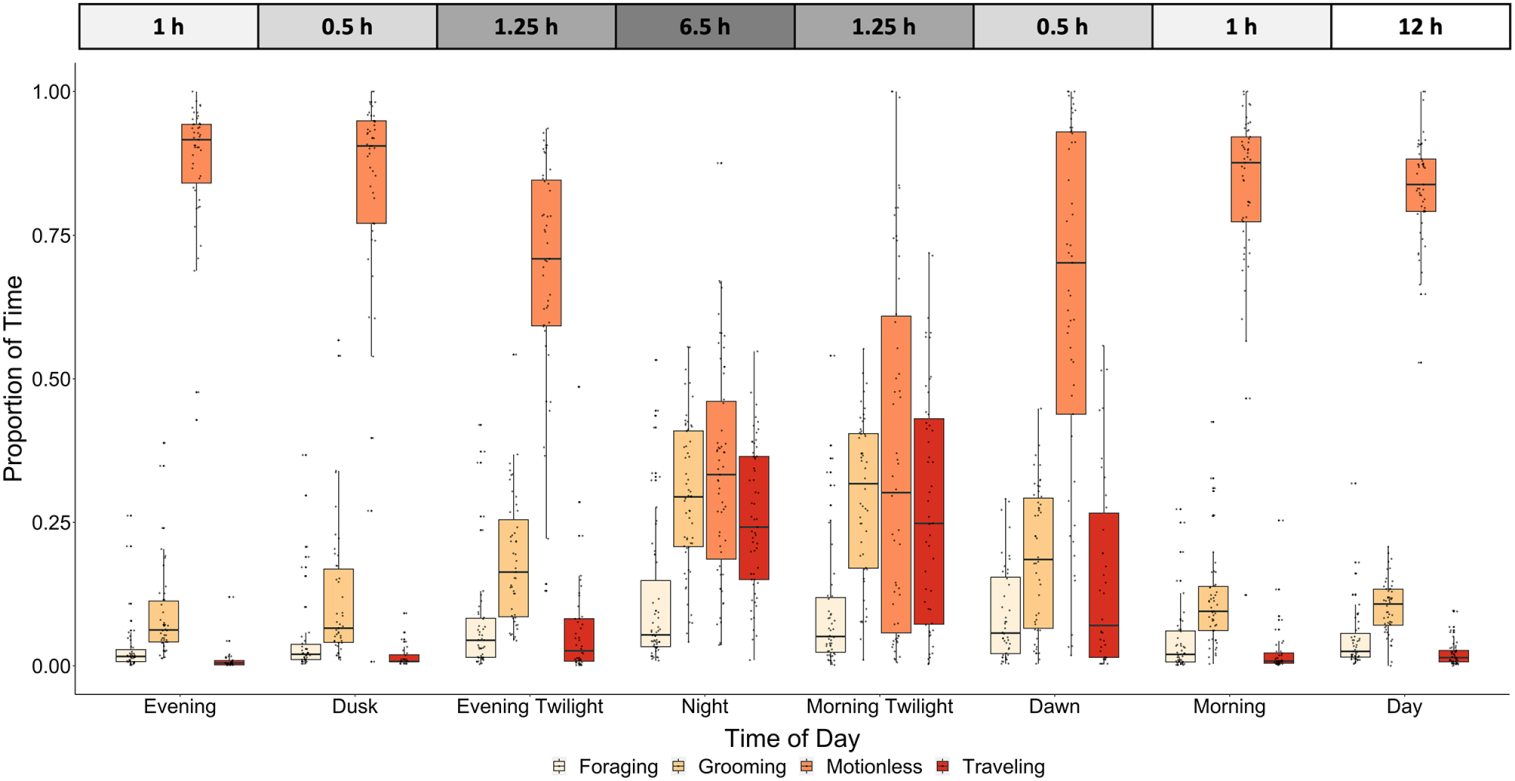
Proportion of time kangaroo rats spent traveling, foraging, motionless and grooming across eight light phases

**Fig. 4:**
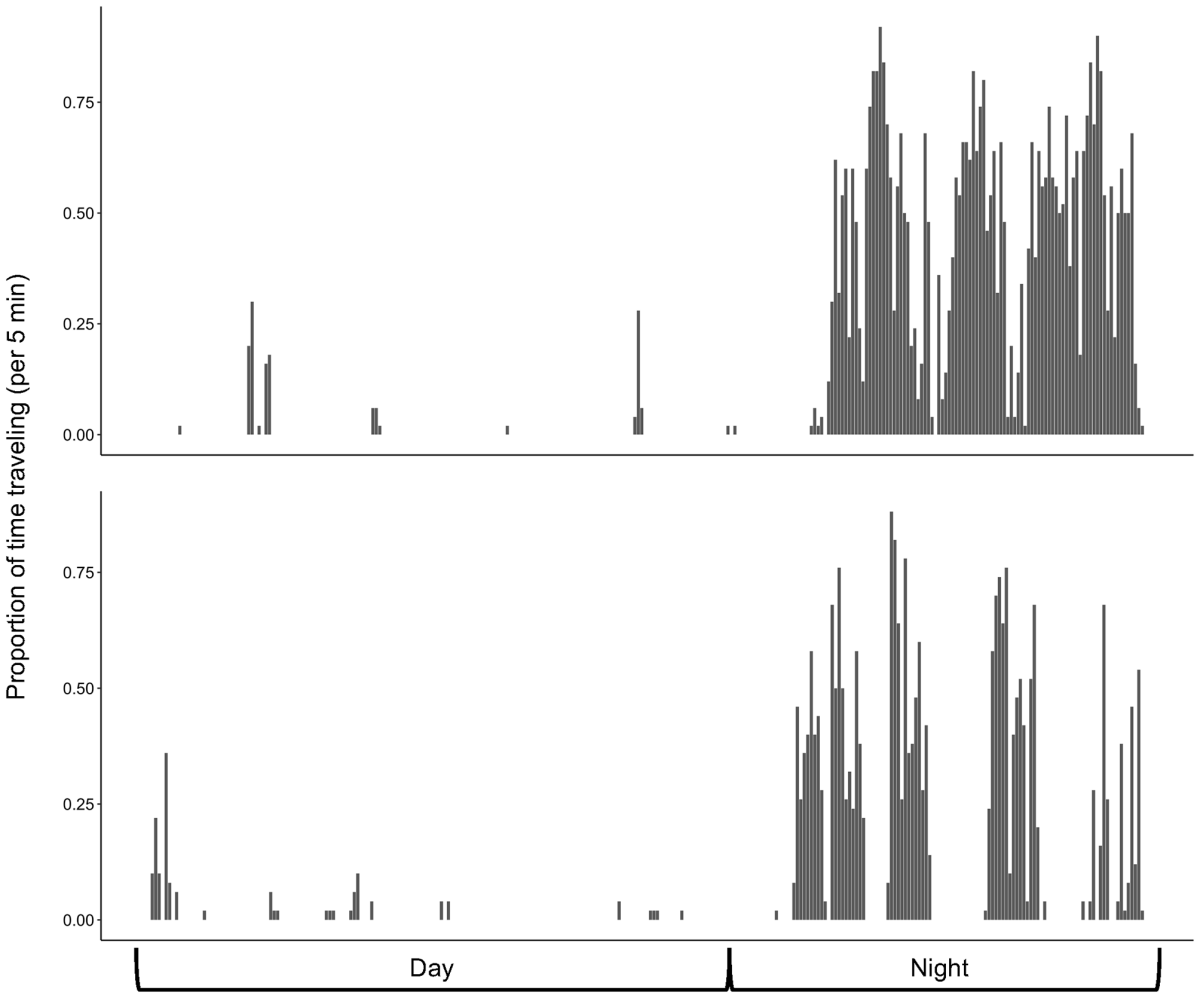
An archetypal 24-hour travel activity cycle for two individuals (top = female; bottom = male) illustrating long periods of non-travel activity during the day with long bouts of traveling at night

By classifying our complete unlabeled free-ranging kangaroo rat accelerometry data set, we confirmed that kangaroo rats are nocturnal, as they spend less time motionless during nighttime light phases (when the sun is below the horizon) compared to daytime light phases (when the sun is above the horizon) and they rarely travel at all during daytime light phases (Fig. 3). Thus, the typical diel activity cycle of a Merriam’s kangaroo rat consisted of long periods of stillness during the day, with very occasional short bouts of activity, followed by long bouts of activity during the night, punctuated by occasional periods of stillness (Fig. 4).

To further understand the proportion of time kangaroo rats spent performing each behavior, we investigated differences across nighttime light phases (i.e., portion of the diel cycle where sun is below the horizon). The proportion of time spent in each behavioral state during different nighttime light phases differed significantly (traveling: *χ*^*2*^ = 397.82, df = 7, P < 0.001, model R^2^_(c)_ = 0.60; motionless: *χ*^*2*^ = 470.31, df = 7, P < 0.001, model R^2^_(c)_ = 0.58; foraging: *χ*^*2*^ = 68.34, df = 7, P < 0.001, model R^2^_(c)_ = 0.47; and grooming *χ*^*2*^ = 267.13, df = 7, P < 0.001, model R^2^_(c)_ = 0.51 ). Interestingly, kangaroo rats spent significantly more time traveling during night and morning twilight compared to all other nighttime light phases (P < 0.01 for all comparisons). Additionally, kangaroo rats spent more time traveling at dawn compared to all other nighttime light phases except for night and morning twilight (P < 0.01 for all comparisons). Thus, although the sun is below the horizon, kangaroo rats are not traveling as much at the beginning of the night (dusk and evening twilight) compared to the mirrored light phases at the end of the night (morning twilight and dawn). We found a similar pattern when examining motionless behavior in kangaroo rats across light phases. Kangaroo rats spent significantly less time motionless at night and morning twilight compared to all other light phases (P < 0.01 across all comparisons). Moreover, kangaroo rats spent significantly more time motionless during evening twilight and dawn compared to morning twilight and night (P < 0.01 across both comparisons), but significantly less time motionless during evening twilight and dawn compared to morning, day, evening, and dusk (P < 0.01 across all comparison). Similarly, kangaroo rats spent significantly more time foraging during night, morning twilight, and dawn compared to all other light phases (P < 0.01 across all comparisons). Lastly, kangaroo rats spent more time grooming during evening twilight, night, morning twilight, and dawn compared to all other light phases (P < 0.01 across all comparisons). Corroborating other activity patterns, kangaroo rats spent significantly more time grooming at night and morning twilight compared to all other light phases (P < 0.01 across all comparisons).

### Behavioral responses to moonlight and environmental variables

**Fig. 5:**
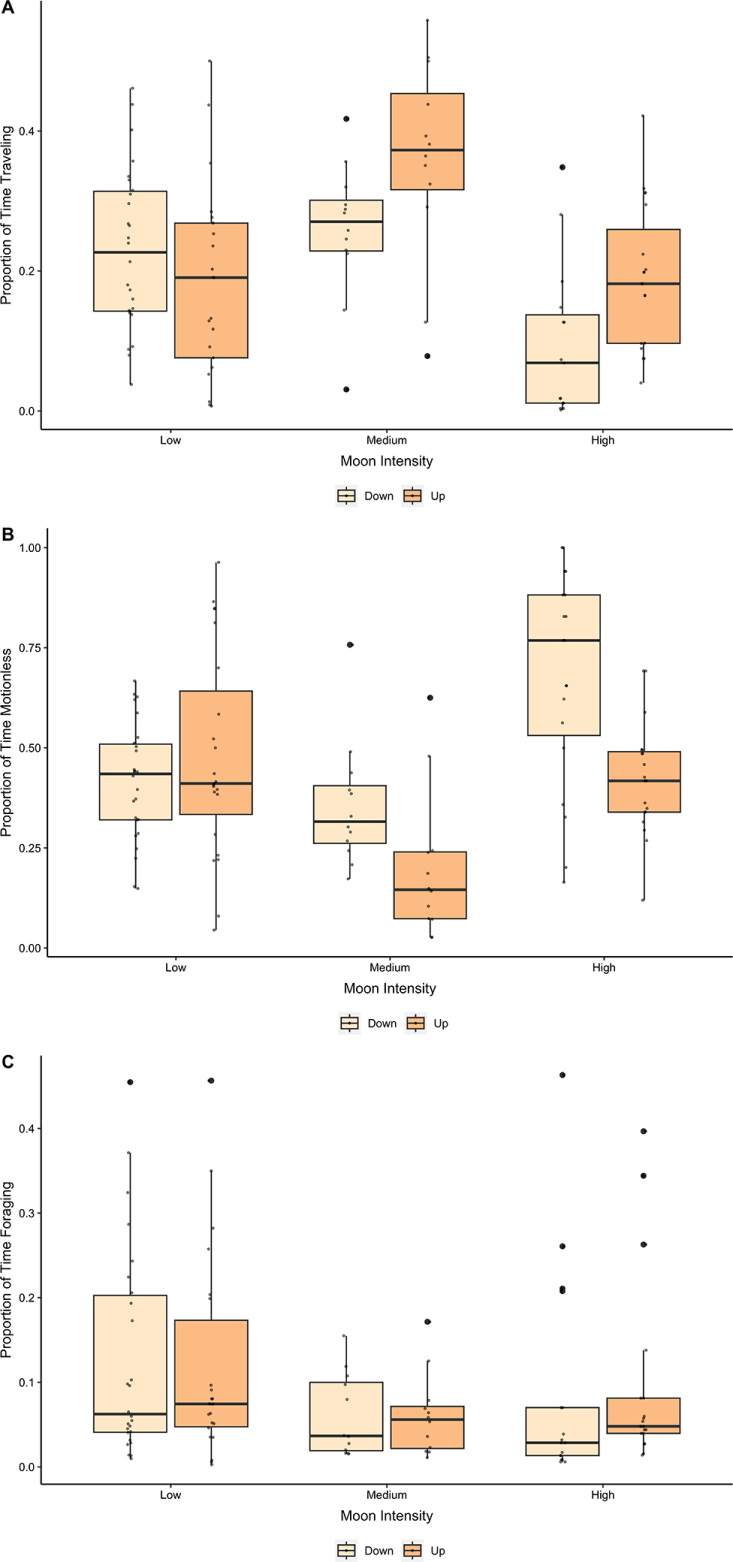
Proportion of time kangaroo rats spent traveling (**A**), motionless (**B**), foraging (**C**), and grooming (**D**) during different levels of moon intensity (Low < 0.33; Medium = 0.33–0.66; High > 0.66) and the impact of moon position (up/risen or down/set)

We examined if the proportion of time spent motionless, traveling, foraging, and grooming was influenced by moon intensity and moon position. When we removed all nights (n = 4) with a precipitation rate > 0 to account for cloud cover, no relationships changed—thus we retained all values in the dataset. For the proportion of time kangaroo rats spent traveling, we found that moon intensity and moon position had no impact on their own (*χ*^*2*^ = 5.42, df = 2, P = 0.07 and *χ*^*2*^ = 2.01, df = 1, P = 0.16, respectively; model R^2^_(c)_ = 0.50), but their interaction was significant (*χ*^*2*^ = 13.33, df = 2, P = 0.001, R^2^_(c)_ = 0.50). Kangaroo rats typically traveled more during medium moon intensity nights and when the moon was visible (Fig. 5A). Moon position (*χ*^*2*^ = 6.34, df = 1, P = 0.01, R^2^_(c)_ = 0.52) and the interaction between moon position and moon intensity (*χ*^*2*^ = 17.48, df = 2, P < 0.001, R^2^_(c)_ = 0.52) significantly impacted the proportion of time kangaroo rats spent motionless. When the moon was visible, kangaroo rats spent significantly less time motionless, and they spent less time motionless during a medium moon intensity compared to high and low moon intensities (Fig. 5B). Moon intensity, moon position, and their interaction had no impact on the proportion of time kangaroo rats spent foraging (*χ*^*2*^ = 5.36, df = 2, P = 0.07, *χ*^*2*^ = 0.40, df = 1, P = 0.53, and *χ*^*2*^ = 1.30, df = 2, P = 0.53, respectively; model R^2^_(c)_ = 0.85; Fig. 5C). Time spent grooming was significantly greater when the moon was visible (*χ*^*2*^ = 11.5575, df = 1, P < 0.01); however, there was no effect of moon intensity and the moon position/moon intensity interaction on the proportion of time spent grooming (*χ*^*2*^ = 2.28, df = 2, P = 0.31, and *χ*^*2*^ = 3.45, df = 2, P = 0.18, respectively; model R^2^_(c)_ = 0.65; Fig. 5D).

Additionally, we examined the proportion of time kangaroo rats spent traveling, motionless, grooming, and foraging by directly comparing each behavior as a function of temperature, relative humidity, wind speed, and moon illumination. We found that kangaroo rats would spend more time foraging as relative humidity increased (*χ*^*2*^ = 4.19, df = 1, P = 0.04, R^2^_(c)_ = 0.63), but other behaviors were not influenced by humidity. Temperature, wind speed, and moon illumination had no effect on the proportion of time kangaroo rats spend foraging, traveling, grooming, or motionless (Additional File 1; Table S5).

## Discussion

By combining low-mass, miniaturized animal-borne accelerometers with radiotelemetry and advanced machine learning techniques, our study showed that accelerometers can be effectively used to quantify behavioral activity of small-bodied (< 50 g), nocturnal, free-ranging mammals. Our work represents the first continuous detailed quantitative description of fine-scale behavioral activity budgets in kangaroo rats, a diverse group of highly abundant rodents that are widely recognized as keystone species in arid regions of North America. After obtaining highly accurate models for ecologically relevant behaviors such as foraging and traveling, we were able to assess behavioral responses to diel light phases, moonlight, ambient air temperature, average windspeed, and average relative humidity. We confirmed that kangaroo rats are highly nocturnal, but are also much more active in some phases of the night cycle than others. Somewhat surprisingly, although we found that behavioral activity cycles are largely independent of several major abiotic factors (most notably moonlight), kangaroo rats did show a significant increase in time spent foraging on more humid nights, an abiotic factor that has received only limited attention in the ecological literature on heteromyid rodents.

### Merriam’s kangaroo rats are not lunarphobic

Much of the ecological literature on nocturnal small mammals indicates that greater moon illumination leads to decreased surface activity and foraging behavior as a result of increased predation risk [58–61]. However, other studies of nocturnal small mammals have indicated both lunarphilia and lunarphobia in regards to foraging/activity patterns, and sometimes no relationship at all [62]. Prugh and Golden’s [62] synthesis on moonlight and predation risk across nocturnal mammals found mixed results, and although the meta-analysis showed that moonlight suppressed activity of nocturnal mammals overall, moonlight effects were not related to trophic level and could be better explained by visual acuity, habitat cover, and phylogenetic relatedness. Mixed results have also been reported for kangaroo rat (*Dipodomys* spp.) behavioral responses to moonlight (e.g., lunarphobia: [36–38, 40]; lunarphilia: [39, 41]; and no influence: [63]). Because previous studies relied on either limited direct observations (i.e., occasional telemetry fixes) or indirect measures (i.e., trapping data and visual encounter surveys), some of the variation could be related to methodological constraints rather than ecological differences.

Our study is the first to use animal-borne biologging devices, which are arguably the most comprehensive and least invasive tools for measuring activity cycles of kangaroo rats, and we found that increased moonlight did not strongly influence the foraging and traveling patterns of *D. merriami*. However, we noted some lunarphilic tendencies. In particular, we found that kangaroo rats spent more time traveling and less time motionless when the moon was up during intermediate moon phases (i.e., medium moon intensity), indicating a preference for activity during the lighter, rather than darker, periods of these nights (corroborating with evidence from Lockard and Owings [39] and Prugh and Brashares [41]) that kangaroo rat species are somewhat lunarphilic). Our data indicate that kangaroo rats generally accept this tradeoff, increasing surface activity under conditions when their own heightened visual acuity [64] would enhance their ability to avoid predatory attacks despite them being more visible to predators. Interestingly, we also found that kangaroo rats do not reduce overall activity on either full moon or new moon nights, indicating that they are still willing to travel and forage under a number of different light conditions—but if they have the option (i.e., the moon is up for only part of the night), they favor traveling with some moonlight available rather than none.

This pattern is in contrast to the findings of Daly et al. [36], who used periodic fixes of radiotagged animals to show that *D. merriami* would make compensatory shifts in foraging behavior on nights with higher moon illumination by foraging at dawn. Our accelerometry data shows that individuals were more active at dawn than other light phases of the day (i.e., morning, day, evening, and dusk) regardless of moon phase and that, when variation in moonlight is available (i.e., during medium moon intensity phases), they prefer to travel more when the moon is visible. However, our study took place during the summer period (May-August), whereas Daly et al. [36] analyzed activity patterns in the winter (December-March), and thus further research would be needed to elucidate any potential interactions between seasonality and lunarphobia. The limited data regarding seasonal ecology of *D. merriami* indicates that this species is generally active throughout the year, but does shift toward a diet consisting mostly of cached seeds in hotter and drier periods when green vegetation is not available [65]—a shift in foraging ecology that could have consequences for how individuals respond to ambient moonlight and predation risk.

Other studies have also concluded that kangaroo rats avoid moonlight, using various methods across different species (i.e., *Dipodomys merriami*, *D. ordii*, *D. panamintinus*, *D. microps*, [40]; *D. spectabilis*, [38]; and *D. ordii*, [37]), including live trapping, recording with feeding stations, and direct observations and counts while driving along dirt and gravel roads. The methods used in these past studies are generally more indirect or less comprehensive than animal-borne accelerometry, which allows for continual quantification of behavioral states across many individuals and days. For example, human observers using radiotelemetry to locate individuals could influence the results of measuring activity by causing kangaroo rats to hide in their burrows when an observer approaches, live-trapping assumes that all animals that are active on the surface are caught, recording with feeding stations provides a sudden and easily accessible food source for kangaroo rats that could alter their motivation for surface activity, and cruising on roads relies on kangaroo rats traveling adjacent to a potentially dangerous loud and fast moving object. However, using accelerometry to quantify traveling and foraging behavior does not provide details of microhabitat use. Although kangaroo rats do not reduce their surface activity with respect to moonlight, they could shift to using more or less vegetative cover during different lighting conditions. Although we cannot address this possibility, future studies using accelerometry may be able to validate more specific categories of kangaroo rat movement, including generating models that could accurately categorize movement in different habitat types. If movement differs consistently among habitat types (for example, slower and more steady movement profiles in heavily vegetated microhabitat compared to moving across open terrain), overall acceleration signatures for movement through these habitats could be accurately classified. The addition of temperature data logged simultaneously with movement by animal-borne devices could also potentially add information about microhabitat use for habitats that have very different temperature profiles (e.g., underground in burrow system versus on the surface).

### Merriam’s kangaroo rats increase foraging effort on humid nights

We expected other major abiotic factors, such as temperature, wind speed, and humidity to potentially influence activity cycles of kangaroo rats (we were unable to examine precipitation due to the very limited rain occurring at our study site). Although temperature and wind speed did not influence activity cycles, increased foraging was associated with higher levels of humidity. In general, kangaroo rats are highly adapted to arid, desert-like conditions and are thought to select microclimates that avoid thermal extremes and evaporative water loss [66–68]. Past studies investigating the suppression of evaporative water loss in relation to humidity have mainly focused on the differences between burrow and surface humidity, and the relationship of surface activity to surface relative humidity has been largely overlooked (but see Tracy & Walsberg, [69]). Kangaroo rats lose most of their water through cutaneous evaporative water loss [69], and so they may conserve water by prioritizing longer foraging times on nights with higher surface humidity. Future ecological studies of kangaroo rats should more carefully consider the role of ambient humidity in driving patterns of movement and activity.

### Merriam’s kangaroo rats are more active prior to dawn than after dusk

Our analyses of acceleration data to determine diel activity patterns of kangaroo rats confirmed that they are exclusively nocturnal. We relocated individuals using radiotelemetry at least once a day during daylight hours, and because we never observed kangaroo rats outside their burrow systems, we assume that the limited daytime movement logged by accelerometry devices largely represents fossorial activity. This corroborates other studies that used direct observation via radio-telemetry and determined almost exclusively nocturnal/crepuscular activity in kangaroo rats ( [70–74]; but see Boal & Giovanni, [33]). Because accelerometers allow for continuous recording of activity and behavior, we were also able to examine much more fine scale activity levels and, in doing so, we found that kangaroo rats are most active during the latter, cooler portions of the night (i.e., the night, morning twilight, and dawn light phases) compared to the beginning and hotter portions of the night (i.e., dusk and evening twilight). Although there is general consensus in the literature that kangaroo rats are almost exclusively nocturnal with few anecdotal accounts of diurnal activity, there is more variability in estimates of activity across different phases of the night cycle [37, 63, 70, 75–77].

Previous studies typically used either observations of captive individuals, or periodic telemetry relocations to estimate activity levels. The most robust study on *Dipodomys* spp. nightly activity patterns to date was done by Langford [75], using direct observation of a focal individual with a red-cellophane covered spotlight throughout the entire night, a time-intensive method that may still have impacted natural behavior from continuous human presence. In contrast, accelerometers in our study continually measured behavior across individuals for up to 6.5 days without the need for direct human presence beyond the collection of a validation dataset.

Our study also provides new insight into the nocturnal activity patterns of Merriam’s kangaroo rat as other studies have shown that *D. merriami* is primarily active at dusk and only three to four hours into the night in the winter months [78], and nocturnal activity in captivity peaked right after and right before sunrise and sunset [76]. In contrast, we found that *D. merriami* were more active later in the night during the summer months. A number of factors could explain this shift in activity, including seasonal availability of resources, temperature, and predation risk. Ectothermic nocturnal predators such as rattlesnakes (three species of rattlesnakes, *Crotalus atrox, C. viridis*, and *C. scutulatus* were all abundant at our site) are warmer and more active (and thus likely more dangerous) during the early portion of the night when the temperature is highest. Additionally, ectothermic predators are a seasonal impact, as they are typically inactive during the winter months throughout *D. merriami’s* range (Hanscom and Clark, unpublished data). Additional research could tease apart the interactive influence of temperature, predation, and seasonality on kangaroo rat activity patterns.

### Free-ranging validation techniques

A critical step in using animal-borne accelerometry to quantify difficult-to-observe behaviors is to validate with time-matched and paired datasets of behavioral observations and acceleration data. Many methods have been developed to conduct validation studies, including independent captive studies [22, 79], observing animals in zoo settings [80], using surrogate species [81], and observations of free-ranging animals [24]. Captive validation studies and the use of surrogate species often have more limited utility because the behaviors observed may not show the same acceleration signatures as free-ranging individuals [81]. Additionally, validation studies (whether in captivity or not) using direct observations by humans could alter the behavior of the focal individual, leading to a lack of species-typical behavior in the validation set. Here, we developed a method to create validation datasets that overcome some of these critical limitations. We used only free-ranging individuals, and we relocated focal individuals by attaching miniature (< 1 g) radio transmitters to the accelerometry device. Because our initial presence sometimes caused individuals to enter burrow systems, upon relocating focal animals, we moved away from their immediate vicinity or the vicinity of their burrow system and used a telephoto video camera with IR recording capability and an IR flashlight to video record behaviors of kangaroo rats at a distance over 10 m. We found that kangaroo rats largely resumed natural activity after human observers were more than ~ 10 m away and made no loud sounds, suggesting the observers could not be detected by the kangaroo rats. Although we typically observed individuals for a relatively short amount of time (i.e., 5–10 min) using this method, we attained sufficient validation data for training models by accumulating multiple observations across individuals and nights. To our knowledge, this is the first time an attempt has been made to fully validate a large accelerometry dataset using only observations of free-ranging and nocturnal individuals in situ.

## Conclusions

Here, we present the first study using accelerometry to quantify behavior on a free-ranging population of a heteromyid rodent, a keystone species in desert areas across North America (Meriam’s kangaroo rat, *Dipodomys merriami*). We provide a proof-of-concept approach using combined accelerometry/telemetry devices to record validation videos and assemble large acceleration datasets that allowed us to quantify behavioral patterns across the summer active period. Our study represents one of the most robust behavioral datasets of a keystone species that is estimated to be the most abundant vertebrate in many habitats across southwestern North America [26]. We found that, in summer months Merriam’s kangaroo rats are only active during the nighttime phases of the diel cycle (confirming exclusive nocturnal/crepuscular activity) and are particularly active during later light phases of the night (i.e., late night, morning twilight, and dawn). Additionally, we found little indication that basic activity cycles are impacted by moonlight, but kangaroo rats would spend more time foraging on nights with higher surface relative humidity. Prior studies on the behavioral responses of kangaroo rats to moonlight and weather have been mixed within and across taxa, and our study highlights the utility of using accelerometry methods to address these questions with large datasets that quantify behavioral details of multiple individuals across long time scales while minimizing human impact. Thus, we hope that our study will motivate researchers to adapt and apply accelerometry using a greater diversity of species, as the approaches for using this *next generation natural history* biologging technique continues to be further developed and improved.

### Electronic supplementary material

Below is the link to the electronic supplementary material.

Supplementary Material 1

## Data Availability

The full data sets used and/or analyzed during the current study are available at: Hanscom, Ryan et al. (2023). Cryptic behavior and activity cycles of a small mammal keystone species revealed through accelerometry: a case study of Merriam’s kangaroo rats [Dataset]. Dryad. 10.6086/D1SQ4Z.

## List of Abbreviations

VHF: Very High Frequency
IR: Infrared
GLMM: Generalized linear mixed modeling
