# Supplementary material for: Cryptic behavior and activity cycles of a small mammal keystone species revealed through accelerometry: a case study of Merriam’s kangaroo rats (Dipodomys merriami)
Source: Mov Ecol. 2023 Nov 2;11:72. doi: 10.1186/s40462-023-00433-x (PMC10621205; doi:10.1186/s40462-023-00433-x)
Supplement: Supplementary file 1 — Supplementary Material 1 [file 40462_2023_433_MOESM1_ESM.docx]

**Supplemental Information**

Figure S1. Initial ethogram used to score behaviors of free-ranging kangaroo rats with an accelerometer packaged attached to their dorsum.


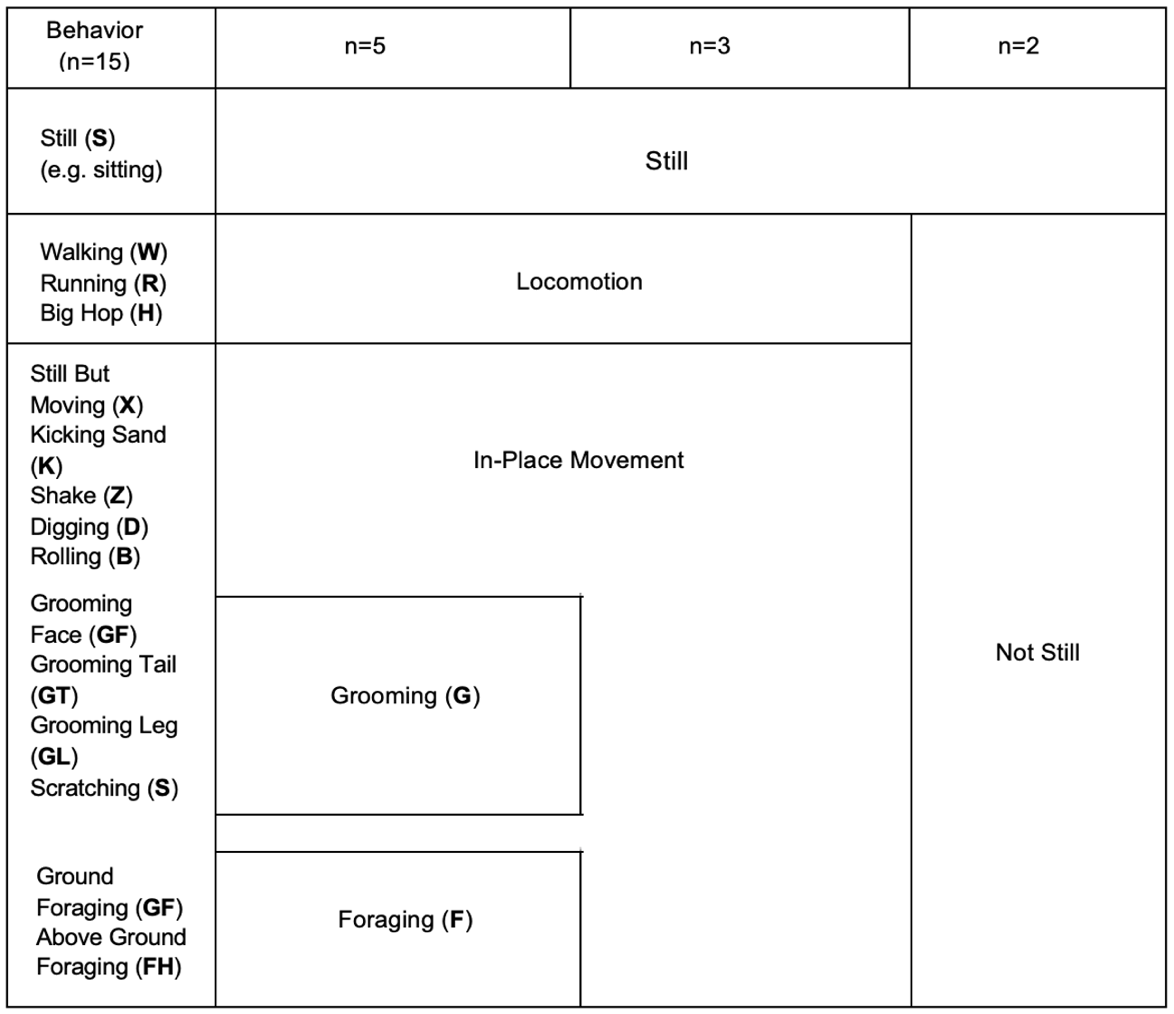


Table S1. Morphometrics of *Dipodomys merriami* that were fitted with accelerometer/VHF biologging units during the summer of 2021 at Marathon Grasslands Preserve, TX USA (SAL = snout-anus length; TL = tail length; and HFL = hind-foot length).

| ID | Sex | Mass (g) | SAL (mm) | TL (mm) | HFL (mm) |
| --- | --- | --- | --- | --- | --- |
| R959 | F | 42 | 75 | 151 | 34 |
| R946 | F | 46 | 88 | 118 | 35 |
| R309 | F | 46 | 78 | 134 | 42 |
| R311 | F | 41 | 64 | 152 | 33 |
| R307 | F | 47 | 93 | 147 | 37 |
| R057 | F | 43 | 65 | 130 | 34 |
| R901 | F | 47 | 86 | 154 | 34 |
| R053 | F | 47 | 81 | 144 | 34 |
| R849 | F | 40 | 61 | 115 | 30 |
| L316 | M | 43 | 83 | 140 | 35 |
| L198 | M | 48 | 79 | 135 | 34 |
| L948 | M | 42 | 95 | 152 | 37 |
| L315 | M | 45 | 90 | 151 | 32 |
| L951 | M | 46 | 93 | 150 | 34 |

Table S2. Observational data collected from free-ranging Merriam’s kangaroo rats (*Dipodomys merriami*) at Marathon Grasslands Preserve in West Texas from May – August 2021. Individuals refers to the total number of different individuals contributing to the validated observational video.

| Behavior | Observation Seconds | Individuals |
| --- | --- | --- |
| Motionless | 4,110 | 8 |
| Foraging | 2,658 | 7 |
| Grooming | 360 | 7 |
| Traveling | 216 | 7 |

Table S3. Model selection results for the behaviors foraging, grooming, traveling and motionless. The top model and distribution family (in bold) for each behavior was used in our GLMM modeling framework (see methods). Akaike Information Criterion (AIC) and AIC weights are provided. Infinite = the distribution family exceeded a very large value and thus the family is not a suitable fit for the data. Error (PIRLS) = Error (Pearson Iterative Residuals for Least Squares) loop encountered a situation that led to a NaN (Not-a-Number) value, which is typically an undefined mathematical value and suggests that the family is not a good fit for the data.

| Behavior | Family | AIC | AIC Weight |
| --- | --- | --- | --- |
| Foraging |  |  |  |
|  | **Gaussian** | **-284.45** | **1.00** |
|  | Binomial | 35.22 | 0.00 |
|  | Poisson | Infinite | 0.00 |
|  | Gamma | Error (PIRLS) | - |
| Grooming |  |  |  |
|  | **Gamma** | **-204.77** | **1.00** |
|  | Gaussian | -176.76 | 0.00 |
|  | Binomial | 63.30 | 0.00 |
|  | Poisson | Infinite | 0.00 |
| Traveling |  |  |  |
|  | **Gamma** | **-165.32** | **1.00** |
|  | Gaussian | -70.30 | 0.00 |
|  | Binomial | 84.19 | 0.00 |
|  | Poisson | Infinite | 0.00 |
| Motionless |  |  |  |
|  | **Gamma** | **63.81** | **1.00** |
|  | Gaussian | 84.61 | 0.00 |
|  | Binomial | 229.53 | 0.00 |
|  | Poisson | Infinite | 0.00 |

Table S4. Accuracy, precision, and recall values obtained for the four behavioral classes across three different algorithms per window size using only M, SD, and ODBA as summary statistics or all summary statistics available on the open access application Accelerater. The random forest model developed at a 6 s window size with all summary statistics included was retained and is in bold. M = mean acceleration; SD = standard deviation, and ODBA = overall dynamic body acceleration. Weighted average and overall percent correct is also given.

| Summary Statistics | Window Size (s) | Algorithm | Classification Performance | Foraging | Grooming | Still | Walking | Weighted Average | % Correct |
| --- | --- | --- | --- | --- | --- | --- | --- | --- | --- |
| M;SD; ODBA | 1 | Linear SVM | Accuracy | 77.39 | 76.61 | 96.2 | 88.07 | 84.57 | 69.13 |
|  |  |  | Precision | 57.99 | 53.49 | 90.05 | 77.56 | 69.77 |  |
|  |  |  | Recall | 46.72 | 64.87 | 94.51 | 72.61 | 69.68 |  |
|  |  | Decision Tree | Accuracy | 78.11 | 78.11 | 96.13 | 86.57 | 84.73 | 69.46 |
|  |  |  | Precision | 57.31 | 57.65 | 94.46 | 71.76 | 70.29 |  |
|  |  |  | Recall | 61.36 | 54.1 | 89.01 | 75 | 69.87 |  |
|  |  | Random Forest | Accuracy | 81.13 | 80.54 | 96.79 | 87.68 | 86.53 | 73.07 |
|  |  |  | Precision | 66.77 | 61.1 | 92.68 | 72.6 | 73.29 |  |
|  |  |  | Recall | 54.29 | 65.64 | 93.96 | 80.32 | 73.55 |  |
| All* | 1 | Linear SVM | Accuracy | 78.77 | 80.14 | 96.59 | 89.06 | 86.14 | 72.28 |
|  |  |  | Precision | 58.52 | 60.86 | 90.84 | 78.65 | 72.21 |  |
|  |  |  | Recall | 55.18 | 61.95 | 95.33 | 78.04 | 72.63 |  |
|  |  | Decision Tree | Accuracy | 77.46 | 76.67 | 96.07 | 85.71 | 83.98 | 67.96 |
|  |  |  | Precision | 55.07 | 54.76 | 88.97 | 72.53 | 67.83 |  |
|  |  |  | Recall | 59.07 | 48.84 | 95.33 | 70.28 | 68.38 |  |
|  |  | Random Forest | Accuracy | 81.85 | 80.8 | 96.79 | 88.4 | 86.96 | 73.92 |
|  |  |  | Precision | 66.47 | 62.31 | 90.08 | 75.49 | 73.58 |  |
|  |  |  | Recall | 56.99 | 62.47 | 97.25 | 80.36 | 74.27 |  |
| M;SD; ODBA | 2 | Linear SVM | Accuracy | 78.72 | 77.38 | 96.43 | 87.35 | 84.97 | 69.94 |
|  |  |  | Precision | 63.4 | 52.75 | 90.2 | 75.54 | 70.47 |  |
|  |  |  | Recall | 52.72 | 59.26 | 93.88 | 77.65 | 70.88 |  |
|  |  | Decision Tree | Accuracy | 80.06 | 79.91 | 96.13 | 87.35 | 85.86 | 71.73 |
|  |  |  | Precision | 63.89 | 59.57 | 88.54 | 74.23 | 71.56 |  |
|  |  |  | Recall | 62.5 | 51.85 | 94.56 | 80.45 | 72.34 |  |
|  |  | Random Forest | Accuracy | 81.7 | 79.32 | 97.02 | 88.69 | 86.68 | 73.36 |
|  |  |  | Precision | 67.43 | 56.97 | 92.62 | 78.14 | 73.79 |  |
|  |  |  | Recall | 64.13 | 58.02 | 93.88 | 79.89 | 73.98 |  |
| All* | 2 | Linear SVM | Accuracy | 80.36 | 77.68 | 95.09 | 88.54 | 85.42 | 70.83 |
|  |  |  | Precision | 60.51 | 52.41 | 94.12 | 77.85 | 71.22 |  |
|  |  |  | Recall | 57.58 | 61.64 | 87.43 | 74.55 | 70.3 |  |
|  |  | Decision Tree | Accuracy | 79.32 | 77.98 | 94.2 | 86.31 | 84.45 | 68.9 |
|  |  |  | Precision | 57.56 | 53.46 | 92.86 | 71.1 | 68.74 |  |
|  |  |  | Recall | 60 | 53.46 | 85.25 | 74.55 | 68.31 |  |
|  |  | Random Forest | Accuracy | 84.08 | 80.8 | 95.54 | 89.58 | 87.5 | 75 |
|  |  |  | Precision | 70.71 | 58.33 | 95.81 | 75.68 | 75.13 |  |
|  |  |  | Recall | 60 | 66.04 | 87.43 | 85.85 | 74.58 |  |
| M;SD; ODBA | 3 | Linear SVM | Accuracy | 78.35 | 78.94 | 97.83 | 88.58 | 85.93 | 71.85 |
|  |  |  | Precision | 56.3 | 55.56 | 93.94 | 79.39 | 71.3 |  |
|  |  |  | Recall | 53.6 | 57.85 | 97.64 | 77.04 | 71.53 |  |
|  |  | Decision Tree | Accuracy | 79.33 | 79.53 | 97.24 | 88.78 | 86.22 | 72.44 |
|  |  |  | Precision | 57.46 | 56.69 | 93.13 | 83.62 | 72.73 |  |
|  |  |  | Recall | 61.6 | 59.5 | 96.06 | 71.85 | 72.25 |  |
|  |  | Random Forest | Accuracy | 83.46 | 84.65 | 97.83 | 90.75 | 89.17 | 78.35 |
|  |  |  | Precision | 65.65 | 69.03 | 93.28 | 83.85 | 77.95 |  |
|  |  |  | Recall | 68.8 | 64.46 | 98.43 | 80.74 | 78.11 |  |
| All* | 3 | Linear SVM | Accuracy | 78.94 | 82.48 | 97.64 | 89.76 | 87.2 | 74.41 |
|  |  |  | Precision | 64.42 | 60.78 | 94.4 | 79.37 | 74.74 |  |
|  |  |  | Recall | 48.91 | 76.23 | 95.93 | 79.37 | 75.11 |  |
|  |  | Decision Tree | Accuracy | 78.94 | 83.07 | 97.44 | 87.8 | 86.81 | 73.62 |
|  |  |  | Precision | 60.42 | 66.67 | 93.65 | 74.62 | 73.84 |  |
|  |  |  | Recall | 63.5 | 59.02 | 95.93 | 76.98 | 73.86 |  |
|  |  | Random Forest | Accuracy | 83.07 | 86.02 | 98.82 | 90.35 | 89.57 | 79.13 |
|  |  |  | Precision | 74.29 | 68.89 | 95.35 | 77.7 | 79.06 |  |
|  |  |  | Recall | 56.93 | 76.23 | 100 | 85.71 | 79.72 |  |
| M;SD; ODBA | 6 | Linear SVM | Accuracy | 82.69 | 85.26 | 98.08 | 90.38 | 89.1 | 78.21 |
|  |  |  | Precision | 66.67 | 63.27 | 100 | 87.88 | 79.45 |  |
|  |  |  | Recall | 61.54 | 86.11 | 92.68 | 72.5 | 78.21 |  |
|  |  | Decision Tree | Accuracy | 80.77 | 82.69 | 99.36 | 89.74 | 88.14 | 76.28 |
|  |  |  | Precision | 63.64 | 60 | 97.62 | 83.33 | 76.15 |  |
|  |  |  | Recall | 53.85 | 75 | 100 | 75 | 75.96 |  |
|  |  | Random Forest | Accuracy | 85.9 | 87.18 | 100 | 92.31 | 91.35 | 82.69 |
|  |  |  | Precision | 79.31 | 68.18 | 100 | 83.33 | 82.71 |  |
|  |  |  | Recall | 58.97 | 83.33 | 100 | 87.5 | 82.45 |  |
| **All*** | **6** | Linear SVM | Accuracy | 83.97 | 85.9 | 97.44 | 92.95 | 90.06 | 80.13 |
|  |  |  | Precision | 59.09 | 81.08 | 92.31 | 91.67 | 81.04 |  |
|  |  |  | Recall | 78.79 | 66.67 | 97.3 | 80.49 | 80.81 |  |
|  |  | Decision Tree | Accuracy | 81.41 | 82.05 | 99.36 | 83.33 | 86.54 | 73.08 |
|  |  |  | Precision | 54.17 | 75.76 | 97.37 | 70.27 | 74.39 |  |
|  |  |  | Recall | 78.79 | 55.56 | 100 | 63.41 | 74.44 |  |
|  |  | **Random Forest** | **Accuracy** | **87.82** | **90.38** | **99.36** | **92.95** | **92.63** | **85.26** |
|  |  |  | **Precision** | **67.5** | **91.67** | **97.37** | **85.71** | **85.56** |  |
|  |  |  | **Recall** | **81.82** | **73.33** | **100** | **87.8** | **87.8** |  |
| M;SD; ODBA | 9 | Linear SVM | Accuracy | 80.56 | 86.11 | 98.61 | 87.5 | 88.19 | 76.39 |
|  |  |  | Precision | 64.71 | 75 | 94.44 | 70.59 | 76.18 |  |
|  |  |  | Recall | 57.89 | 75 | 100 | 75 | 76.97 |  |
|  |  | Decision Tree | Accuracy | 66.67 | 76.39 | 98.61 | 86.11 | 81.94 | 63.89 |
|  |  |  | Precision | 38.1 | 57.89 | 100 | 68.75 | 66.18 |  |
|  |  |  | Recall | 42.11 | 55 | 94.12 | 68.75 | 64.99 |  |
|  |  | Random Forest | Accuracy | 84.72 | 90.28 | 100 | 88.89 | 90.97 | 81.94 |
|  |  |  | Precision | 72.22 | 84.21 | 100 | 72.22 | 82.16 |  |
|  |  |  | Recall | 68.42 | 80 | 100 | 81.25 | 82.42 |  |
| All* | 9 | Linear SVM | Accuracy | 84.72 | 86.11 | 95.83 | 86.11 | 88.19 | 76.39 |
|  |  |  | Precision | 73.33 | 85.71 | 90 | 60.87 | 77.48 |  |
|  |  |  | Recall | 61.11 | 60 | 94.74 | 93.33 | 77.3 |  |
|  |  | Decision Tree | Accuracy | 73.61 | 88.89 | 100 | 76.39 | 84.72 | 69.44 |
|  |  |  | Precision | 46.67 | 83.33 | 100 | 45 | 68.75 |  |
|  |  |  | Recall | 38.89 | 75 | 100 | 60 | 68.47 |  |
|  |  | Random Forest | Accuracy | 81.94 | 91.67 | 100 | 87.5 | 90.28 | 80.56 |
|  |  |  | Precision | 69.23 | 88.89 | 100 | 63.64 | 80.44 |  |
|  |  |  | Recall | 50 | 80 | 100 | 93.33 | 80.83 |  |

* All = the following summary statistic were used when developing the model: mean, standard deviation, skewness, kurtosis, maximum, minimum, vector norm, covariance, Pearson correlation, dynamic body acceleration, overall dynamic body acceleration (ODBA), mean-diff, std-diff, wave amplitude, line crossings, 25 percentile, 50 percentile, 75 percentile.

Table S5. GLMM results used to investigate how the abiotic factors of temperature, wind speed, relative humidity, and moon illumination affected kangaroo rat behavior. We calculated model fit using conditional R-squared values (see results) and significance of fixed effects was determined using Wald chi-square (*χ^2^*) tests. Degrees of freedom (df) and p values are also reported.

| Behavior | Abiotic Factor | *χ^2^* | df | P |
| --- | --- | --- | --- | --- |
| Traveling | Temperature | 0.21 | 1 | 0.64 |
|  | Relative Humidity | 0.66 | 1 | 0.42 |
|  | Wind Speed | 0.09 | 1 | 0.77 |
|  | Moon Illumination  Sex | 0.01  0.05 | 1  1 | 0.97  0.82 |
|  |  |  |  |  |
| Foraging | Temperature | 1.20 | 1 | 0.27 |
|  | **Relative Humidity** | **4.19** | **1** | **0.04*** |
|  | Wind Speed | 1.15 | 1 | 0.28 |
|  | Moon Illumination  Sex | 0.07  0.03 | 1  1 | 0.79  0.97 |
|  |  |  |  |  |
| Grooming | Temperature | 0.02 | 1 | 0.88 |
|  | Relative Humidity | 0.93 | 1 | 0.33 |
|  | Wind Speed | 0.47 | 1 | 0.49 |
|  | Moon Illumination  Sex | 1.51  0.07 | 1  1 | 0.21  0.79 |
|  |  |  |  |  |
| Motionless | Temperature | 0.08 | 1 | 0.78 |
|  | Relative Humidity | 0.75 | 1 | 0.39 |
|  | Wind Speed | 0.06 | 1 | 0.82 |
|  | Moon Illumination  Sex | 0.04  0.27 | 1  1 | 0.84  0.60 |
